# The Promise of Ambient AI Technology in Medical Education: Opportunities and Guardrails

**DOI:** 10.2196/88725

**Published:** 2026-09-08

**Authors:** Shirin Shafazand, Umar Bowers, Sudha Jayaraman

**Affiliations:** 1 Department of Medicine, Division of Pulmonary, Critical Care and Sleep Medicine, Miller School of Medicine, University of Miami, PO Box 016960 (D60), Miami, FL, 33101, United States, 1 3052437888; 2 UNCW College of Health and Human Services, Wilmington, NC, United States; 3 Department of Surgery, University of Utah, Salt Lake City, UT, United States; 4 Suki.AI, Redwood City, CA, United States

**Keywords:** AI, ambient AI, AI scribe, medical education, deskilling, Evidence based practice, UME, GME

## Abstract

Ambient AI technologies, commonly known as AI scribes, are transforming clinical practice by autonomously capturing patient-provider conversations and structuring them into clinical notes. Short-term studies suggest that ambient AI can significantly reduce documentation time and improve job satisfaction. However, as health care systems accelerate the adoption of these tools, the medical education community must seek to answer unresolved questions regarding their full impact on learners. The inevitable integration of ambient AI in teaching hospitals presents both transformative opportunities and significant challenges for medical education. This viewpoint discusses the potential benefits and risks of ambient AI in both undergraduate and graduate medical education and considers its impact on learning and clinical skills acquisition. Robust research studies are urgently needed to navigate this new frontier and ensure that the integration of ambient AI in the clinical learning environment ultimately enriches, rather than diminishes, the practice of medicine.

## Ambient AI and Clinical Care

Operating in the background, ambient AI technologies, commonly known as AI scribes, capture the patient-provider conversation to identify medically relevant information and structure it into a coherent clinical note. A significant contributor to health provider burnout, the administrative burden of clinical documentation in electronic health records (EHRs) is reduced by deploying these tools in the clinical workflow. Early studies evaluating these tools suggest that ambient AI shortens the time required to sign off and close a patient chart encounter, reduces time spent after hours completing notes, improves provider job satisfaction, potentially improves patient engagement during the clinical encounter, decreases short-term provider burnout, and may better address documentation gaps [1-3].

Despite these benefits, questions about the accuracy or quality of ambient AI remain. More research is needed to address several aspects of this technology, including the safety and health impact of omission and commission errors (eg, not including information, providing incorrect contextual interpretation, or adding incorrect content), its utility in certain clinical environments, its ability to adequately understand and transcribe the speech of patients or providers with differing accents [4], the optimal integration and adoption of the technology into clinical workflows and EHRs, its overall cost-effectiveness, and its long-term impact on the cognitive workload and clinical reasoning of even seasoned clinicians [5,6]. Clinical implementation should follow a thoughtful and guided approach, anchored in implementation science and quality improvement pedagogy [7,8].

## Ambient AI and Medical Education

### Overview

There is no doubt, however, that as this technology improves and the health system gains more confidence in its use, there is likely to be widespread adoption of ambient AI, akin to moving away from paper charts and adopting EHRs several decades ago. Medical trainees are likely to witness this change and even want to use these tools themselves during their training. It is therefore imperative for medical educators to pay close attention to how these tools are being introduced and used in medical schools, residencies, and fellowship training programs where the youngest members of the physician workforce are often engaged with and more likely to be early adopters of new technologies in their daily personal and professional lives. Critically, there are few data on the potential use and impact of these technologies on formative educational experiences, such as skill development in clinical communication, reasoning, and documentation.

This viewpoint highlights the challenges that may be caused by offloading documentation during training across the medical education continuum when learners develop reasoning, synthesis, and communication skills. We share potential consequences, outline deliberate guardrails, and advocate for intentional investigation into understanding these risks.

### Undergraduate Medical Education

We have not found any published studies to date that look at the impact of ambient AI on the early acquisition of clinical documentation and reasoning skills. A potential benefit of automating the note-taking process may be that medical students become more present and engaged during patient and standardized patient encounters, allowing for a deeper focus on developing fundamental skills, such as taking histories, performing physical examinations, and building patient rapport. The reduction in students’ documentation cognitive load may allow for more capacity to observe, process, and learn from the nuances of the patient-provider interaction and the clinical reasoning demonstrated by their preceptors. Early exposure to and training with AI tools could better prepare students for the technologically advanced health care environments in which they will eventually practice. Ambient AI could offer complementary educational opportunities as well. By teaching students to critically engage with the notes generated by ambient AI and comparing these notes to the students’ own independently written notes, faculty have an opportunity to initiate meaningful discussions about communication, documentation structure, important details that guide the differential diagnosis, and clinical reasoning. However, educational theory encompasses the process of learning by doing, which is a key value of clinical documentation and reasoning. It emphasizes active learning, deliberate practice, cognitive apprenticeship, and scaffolding, all in the progression from novice to expert. These components of learning may be negatively affected by removing clinical documentation during the undergraduate medical education process.

### Graduate Medical Education

In graduate medical education, small pilot studies of resident physicians across different specialties suggest increased satisfaction and decreased short-term burnout with ambient AI use. The reports vary on the impact that ambient AI scribe tools have on time spent documenting clinical notes both during and outside of work hours [9-11]. In theory, less time on clinical documentation should allow for more time spent on patient care, knowledge acquisition, or practicing new skills. To date, there are no published studies evaluating whether residents or fellows foster more effective relationships with their patients when the burden of clinical documentation is reduced through ambient AI use. Additionally, we have not seen any specific curricula on teaching trainees effective reviewing, editing, and oversight of the output from ambient AI scribes beyond vendor-created technical skills on how to navigate platforms.

## Potential Unintended Consequences and Guardrails

The benefits of ambient AI in clinical practice need to be balanced against the possibility of unintended consequences, which require clear and intentional guardrails to prevent harm (Table 1).

Writing a clinical note is an important exercise in organizing thoughts, identifying knowledge gaps, and communicating effectively with other health care professionals. Overreliance on AI-generated notes may cause these skills to atrophy. Unintended consequences include the potential for diminished development of essential clinical reasoning skills or cognitive deskilling in those who have newly learned these skills. The act of manually documenting a patient encounter nudges trainees to actively synthesize the information they have gathered, formulate a differential diagnosis, and develop a treatment plan. Automating this process could lead to a more passive learning experience and a superficial understanding of the patient’s condition. Ambient AI may be prone to errors of interpretation and to the omission of nuanced details. Faculty will need training on how to effectively teach and supervise students when using ambient AI and when best to introduce these tools.

**Table 1.** Risks of and guardrails for ambient AI use among trainees.

Dimension	Risks specific to setting	Recommended guardrails
Clinical documentation		
UME^a^	Premature automation of summarization skills before competency is established.	Require unaided notes as default; permit AI-drafted notes only after demonstrated competence and only as a comparison exercise.
GME^b^	Erosion of clinical summarization if notes are accepted uncritically; attestation ambiguity.	Use mandatory attestation language, structured side-by-side comparison in early PGY-1^c^, and periodic competency checks.
Ambient AI used for formative feedback on encounters		
UME and GME	Feedback quality varies with rubric design and prompt engineering; AI may miss interpersonal nuance; risk of feedback being treated as summative when intended as formative.	Use validated rubrics; keep feedback formative and separate from grading; faculty evaluate the quality of AI feedback as a pilot before learner rollout.
Clinical reasoning development		
UME and GME	“Cognitive debt” from repeated cognitive offloading; loss of the synthesis step between data gathering and assessment.	Require learners to generate the assessment and plan before viewing any AI draft; mandate periodic AI-free encounters, with frequency determined by training seniority level; explicitly assess reasoning, not just final notes.
Privacy, consent, and surveillance		
UME	Learner anxiety about surveillance; may impact wellness; ambiguous data retention.	Use written learner consent for any educational recording with defined retention and use limits; clearly separate formative and summative use.
UME and GME	Inconsistency in obtaining patient consent.	Teach explicit, repeatable patient consent scripts as a clinical skill.
Equity and algorithmic bias		
UME and GME	AI speech recognition is known to perform worse for some accents and dialects; AI-generated assessments may encode systemic biases if not actively monitored.	Build equity monitoring into deployment from day 1; teach learners to detect and escalate differential performance; require vendor disclosure of training data and performance disaggregated by patient demographics.
Liability and attestation		
UME and GME	Documentation accountability is important; uncritical acceptance of AI drafts is a concerning risk.	Use an institutional attestation policy requiring explicit review of AI drafts, an audit trail of edits, faculty cosignature for any trainee-associated AI-drafted content, and education on accountability and liability.
Workforce AI literacy		
UME and GME	Curricular drift if AI use is not deliberately taught; students may absorb tool use without absorbing critical appraisal.	Define and assess AI literacy competencies at the UME and GME level, including mechanics, failure modes, equity, consent, and attestation, before deploying ambient tools.

a UME: undergraduate medical education.

b GME: graduate medical education.

c PGY-1: postgraduate year 1.

## Value of Evidence-Based Practice

Research on the impact and utility of ambient AI in medical education and the developmental acquisition of skills is needed. Priority should be given to proposing and implementing a pragmatic study that allows for controlled exposure to ambient AI while adjusting for individual variations in learning outcomes. Such a study should use rigorous methodologies, such as multicenter randomized crossover trial or step-wedge or parallel-group designs, to carefully determine the potential for impact on the fundamentals of medical education. Specific educational outcomes of interest need to be studied, such as note quality and clinical reasoning, patient communication, and time spent in direct patient care, as do changes in cognitive load and burnout from use of ambient AI, among other areas of impact. Additionally, vendor neutrality will also have to be considered, though studies may need to be conducted across vendors based on ambient AI features available for study, such as clinical decision support or diagnostic reasoning.

Furthermore, concerns about consent as well as patient and trainee privacy will need to be addressed when such technology is implemented in medical education, especially if audio recording is used to teach trainees and give feedback. Lastly, there is increasing evidence that ambient AI is used differently across specialties; therefore, trainee education will also have to be modified by specialty [12,13]. A one-size-fits-all training plan is unlikely to translate across all specialties, especially for fields such as pediatrics, where clinical practice has specific nuances, such as developmental stage of the patient and complex caregiver relationships.

## Summary

Ambient AI holds the promise of reducing the documentation burden of modern medical practice and offering benefits in both clinical workflow and medical education. The impact of these tools on our medical students, residents, and fellows must be actively studied. Medical schools themselves need guidance on how to incorporate these technologies to the maximal benefit and least harm of their students and trainees. Accrediting agencies such as the Liaison Committee on Medical Education need evidence to set policy on the impact of ambient AI on medical education. We call for a thoughtful and evidence-based approach to realize the potential of ambient AI while mitigating the risks to the acquisition and retention of fundamental skills for the next generation of physicians. Robust research studies are urgently needed to navigate this new frontier and ensure that the integration of ambient AI in the clinical learning environment ultimately enriches, rather than diminishes, the practice of medicine.

## Abbreviations

EHR: electronic health record
GME: graduate medical education
PGY-1: postgraduate year 1
UME: undergraduate medical education
